# Supplementary material for: A Novel Technique for the Generation of Substantial Numbers of Functional Resident T Cells from Kidney Tissue
Source: Cells. 2022 Jul 18;11(14):2233. doi: 10.3390/cells11142233 (PMC9321143; doi:10.3390/cells11142233)
Supplement: Supplementary file 1 [file cells-11-02233-s001.zip › cells-1815011-supplementary.pdf]

Supplementary Table S1 Monoclonal antibodies for characterization of phenotype and function of kidney T cells

Marker	Fluorochrome	Company	Clone
FVS780		BD	none
CD45	APC	BD Pharmingen	HI30
CD3	BV510	Biolegend	UCHT1
CD4	PE	Biolegend	RPA-T4
CD8	PerCP-Cy5.5	Biolegend	RPA-T8
CD56	PE-Cy7	BD Pharmingen	B159
CD69	FITC	Biolegend	FN50
CD103	BV421	Biolegend	Ber-ACT8
CD3	AF700	Biolegend	HIT3a
CD4	PerCP-Cy5.5	BD Pharmingen	RPA-T4
CD8	BV510	Biolegend	RPA-T8
CD103	BV711	Biolegend	Ber-ACT8
CD69	PE	Biolegend	FN50
CD45RA	BV650	Biolegend	HI100
CCR7	BV421	Biolegend	G043H7
CD28	BV785	Biolegend	CD28.2
CD27	BV605	BD Horizon	L128
CD56	BV570	Biolegend	HCD56
CD161	PE-Dazzle594	Biolegend	HP-3G10
FOXP3	AF488	Biolegend	259D
RORγ	APC	Invitrogen	B2D
T-bet	PE-Cy7	Biolegend	4B10
CD69	BV421	BD Horizon	FN50
CD103	APC	Biolegend	Ber-ACT8
TCR tubes		Beckman Coulter	Clone
A Vβ 5.3	PE		3D11
Vβ 3	FITC		CH92
Vβ 7.1	PE + FITC		ZOE
B Vβ 9	PE		FIN9
Vβ 16	FITC		TAMAYA 1.2
Vβ 17	PE + FITC		E17.5F3
C Vβ 18	PE		BA62.6
Vβ 20	FITC		ELL1.4
Vβ 5.1	PE + FITC		IMMU157

Supplementary Table S1 Monoclonal antibodies for characterization of phenotype and function of kidney T cells

D	Vβ 13.1	PE		IMMU222
	Vβ 8	FITC		56C5.2
	Vβ 13.6	PE + FITC		JU74.3
E	Vβ 5.2	PE		36213
	Vβ 12	FITC		VER2.32
	Vβ 2	PE + FITC		MPB2D5
F	Vβ 23	PE		AF23
	Vβ 21.3	FITC		IG125
	Vβ 1	PE + FITC		BL37.2
G	Vβ 11	PE		C21
	Vβ 14	FITC		CAS1.1.3
	Vβ 22	PE + FITC		IMMU546
H	Vβ 13.2	PE		H132
	Vβ 7.2	FITC		ZIZOU4
	Vβ 4	PE + FITC		WJF24
CD107a		FITC	Biolegend	H4A3
IL-2		BV421	BD Horizon	MQ1-17H12
IL-17a		BV785	Biolegend	BL168
IL-6		PE-Cy7	Biolegend	MQ2-13A5
IL-21		AF647	Biolegend	3A3-N2
IFNγ		BV650	Biolegend	4S.B3
TNFα		BV605	Biolegend	Mab11
CD14		APC-Cy7	BD Pharmingen	MΦP9
CD19		APC-Cy7	BD Pharmingen	SJ25C1
CD56		APC-Cy7	BD Pharmingen	HCD56
CD137		APC	BD	4B4-1

Supplementary Table S1 Monoclonal antibodies for characterization of phenotype and function of kidney T cells

Abbreviations:

APC	AlloPhycoCyanin
BV	Brilliant Violet
PE	Phyco-erythrin
PerCP (-Cy5.5)	Peridinin-Chlorophyll Protein complex
FITC	Fluorescein IsoThioCyanate
FVS780	Fixeable Viability Stain-780
AF	Alexa-Fluor

Supplementary Table S2 Lymphocyte composition following direct enzymatic processing of kidney tissue obtained from nephrectomy versus transplantectomy

	Lymphocyte population		*p**	Resident ($CD69^{+}CD103^{+/-}$) (%)		*p**
	nephrectomy	transplantectomy		nephrectomy	transplantectomy	
T cells (cells/mm^3)	186 (37-342)	1269 (379-2258)	0.02	82.94 (67.60-88.94)	93.43 (78.69-98.37)	0.20
$CD4^{+}$T cells (% of CD3+ T cells)	56.06 (45.81-69.71)	28.96 (17.58-49.52)	0.11	86.04 (76.64-91.10)	93.75 (74.47-97.54)	0.49
$CD8^{+}$T cells (% of CD3+ T cells)	35.97 (23.08-47.91)	63.61 (39.44-77.50)	0.11	84.46 (62.31-88.52)	94.17 (85.59-99.12)	0.11
$CD4^{-}CD8^{-}$T cells (% of CD3+ T cells)	5.00 (3.21-5.73)	5.56 (2.78-10.78)	0.89	74.35 (55.56-79.16)	89.38 (83.99-94.76)	0.03
$CD161^{+}$T cells (% of CD3+ T cells)	14.75 (10.80-17.25)	13.65 (6.80-17.69)	0.89	94.88 (87.43-95.19)	98.58 (92.62-99.08)	0.20
NK cells ($CD56^{+}CD3^{-}$) (cells/mm^3)	11 (6-75)	52 (10-106)	0.29	67.01 (59.33-75.10)	87.97 (79.11-94.05)	0.03

Median and IQ range depicted

* The different populations for kidney tissue obtained from nephrectomy (*n*=4) and transplantectomy (*n*=4) were compared using Mann-Whitney tests, $p<0.05$ was considered significantly different.

Supplementary Table S3 CD4$^+$ and CD8$^+$ T cell phenotype characteristics following direct enzymatic processing of kidney tissue obtained from a nephrectomy and transplantectomy

	Resident (CD69$^+$CD103$^{+/-}$) (%)		*p* **
	nephrectomy	transplantectomy	
CD4$^+$T cells			
Naïve (CD45RA+CCR7+)*	1.54 (0.22-2.90)	1.94 (0.23-4.46)	0.69
CM (CD45RA-CCR7+)*	8.33 (6.70-20.18)	24.73 (5.42-46.44)	0.69
EM (CD45RA-CCR7-)*	89.46 (73.18-90.07)	70.63 (47.33-92.21)	0.69
EMRA (CD45RA+CCR7-)*	1.81 (1.06-4.54)	1.99 (1.25-3.38)	1.00
CD27+CD28+*	26.05 (8.64-32.59)	31.46 (11.42-45.64)	0.49
CD27-CD28+*	61.41 (52.36-85.49)	55.89 (42.09-80.44)	0.49
CD27+CD28-*	0.43 (0.09-0.87)	1.20 (0.33-3.55)	0.34
CD27-CD28-*	9.28 (1.92-20.89)	7.42 (6.30-14.27)	0.89
Tregs (CD45RA+/-FOXP3+)*	1.38 (0.76-3.11)	2.31 (1.31-2.42)	0.49
Tbet+*	65.48 (52.94-87.55)	76.47 (68.21-88.21)	0.69
RORγ+*	2.01 (1.84-4.71)	2.92 (1.99-3.72)	0.54
FOXP3+*	4.68 (2.54-6.97)	5.34 (3.87-9.80)	0.69
CD8$^+$T cells			
Naive (CD45RA+CCR7+)*	1.51 (0.81-2.11)	2.65 (0.42-6.28)	0.69
CM (CD45RA-CCR7+)*	4.26 (3.56-6.34)	5.27 (1.54-9.03)	0.89
EM(CD45RA-CCR7-)*	68.94 (48.13-83.17)	80.13 (69.31-92.56)	0.49
EMRA (CD45RA+CCR7-)*	26.41 (8.74-46.04)	11.95 (5.07-15.81)	0.34
CD27+CD28+*	8.97 (0.92-18.12)	36.44 (22.09-53.95)	0.03
CD27-CD28+*	31.73 (6.27-50.84)	13.70 (5.92-19.38)	0.69
CD27+CD28-*	10.94 (0.41-42.82)	29.10 (17.75-43.67)	0.49
CD27-CD28-*	47.97 (32.59-48.42)	17.40 (8.90-31.71)	0.06
Tbet+*	78.01 (72.68-91.34)	88.72 (81.39-91.85)	0.49
RORγ+*	2.79 (1.12-4.44)	1.13 (0.58-1.73)	0.34

FOXP3+*	1.30 (0.88-1.56)	0.43 (0.18-1.15)	0.11

Median and IQ range depicted
* proportions within CD69+CD103+/- (resident) T cells are given
** subset composition was compared for kidney tissue obtained from a nephrectomy (N=4) versus that of a transplantectomy (N=4) using Mann Whitney tests, $p<0.05$ was considered statistically significant

Supplementary Table S4 Proportions of cytokine producing and CD107a-expressing T cells following direct enzymatic processing of renal tissue obtained from a nephrectomy and transplantectomy

	CD103+		*p****	CD103-		*p***
	Nephrectomy (*n*=6)	Transplantectomy (*n*=4)		Nephrectomy (*n*=6)	Transplantectomy (*n*=4)	
CD4+T cells						
CD107a*	15.79 (7.30-23.50)	34.99 (15.75-50.85)	0.07	14.29 (10.94-26.06)	27.42 (19.7-30.50)	0.07
TNF-α*	90.90 (85.63-95.94)	66.37 (54.63-89.52)	0.17	76.71 (67.61-79.51)	53.96 (36.11-60.52)	0.02
IL-2*	69.44 (31.51-83.15)	34.86 (14.73-74.41)	0.35	61.06 (19.65-67.07)	27.41 (11.00-49.19)	0.11
IFN-γ*	75.78 (74.24-81.00)	64.80 (32.45-82.67)	0.17	59.00 (44.00-62.20)	28.99 (15.30-37.41)	0.07
IL-6*	2.62 (1.64-3.82)	1.46 (0.80-3.03)	0.35	2.13 (1.44-2.55)	0.35 (0.30-1.18)	0.02
IL-17A*	4.34 (3.44-5.99)	6.83 (2.60-17.25)	0.48	2.33 (1.16-4.02)	1.86 (1.53-2.21)	0.76
IL-21*	10.53 (8.72-14.51)	15.91 (11.22-33.20)	0.26	4.61 (2.69-9.83)	9.76 (6.31-16.86)	0.11
Polyfunctional *#	82.33 (75.58-89.79)	60.73 (34.11-85.77)	0.17	69.59 (62.14-73.93)	45.96 (23.77-54.49)	0.04
CD8+T cells						
CD107a*	32.75 (17.74-37.77)	71.98 (56.09-89.67)	<0.01	36.31 (22.43-38.82)	60.82 (44.09-65.88)	0.02
TNF-α*	83.88 (73.74-91.51)	21.74 (12.99-51.40)	<0.01	80.79 (74.51-87.44)	41.05 (25.13-63.11)	<0.01
IL-2*	30.10 (14.59-50.89)	3.28 (1.27-31.99)	0.11	29.50 (7.59-44.07)	6.44 (1.73-27.86)	0.26
IFN-γ*	79.05 (62.82-93.44)	56.53 (14.34-79.44)	0.26	70.68 (40.73-82.66)	48.95 (21.87-64.75)	0.26
IL-6*	3.16 (1.53-4.19)	0.65 (0.23-1.38)	0.02	1.71 (0.52-2.30)	0.29 (0.17-0.69)	0.04
IL-17A*	3.29 (2.06-6.47)	0.84 (0.38-3.21)	0.11	1.14 (0.30-1.57)	0.17 (0.05-0.29)	0.03
IL-21*	2.64 (1.58-4.86)	2.08 (0.91-6.89)	0.61	1.75 (0.97-2.55)	1.83 (1.03-3.08)	0.97
Polyfunctional *#	77.77 (65.15-87.94)	53.52 (17.46-68.29)	0.04	79.69 (54.66-84.39)	56.84 (25.42-68.55)	0.17

Median and IQ range depicted
* proportions within CD103+/- T cells, respectively
# polyfunctional defined as ≥2 pro-inflammatory and/or degranulation factors (TNF-α, IL-2, IFN-γ and/or CD107a)/ cell

**** The different populations for kidney tissue obtained from nephrectomy and transplantectomy were compared using Mann-Whitney tests, *p*<0.05 was considered significantly different.

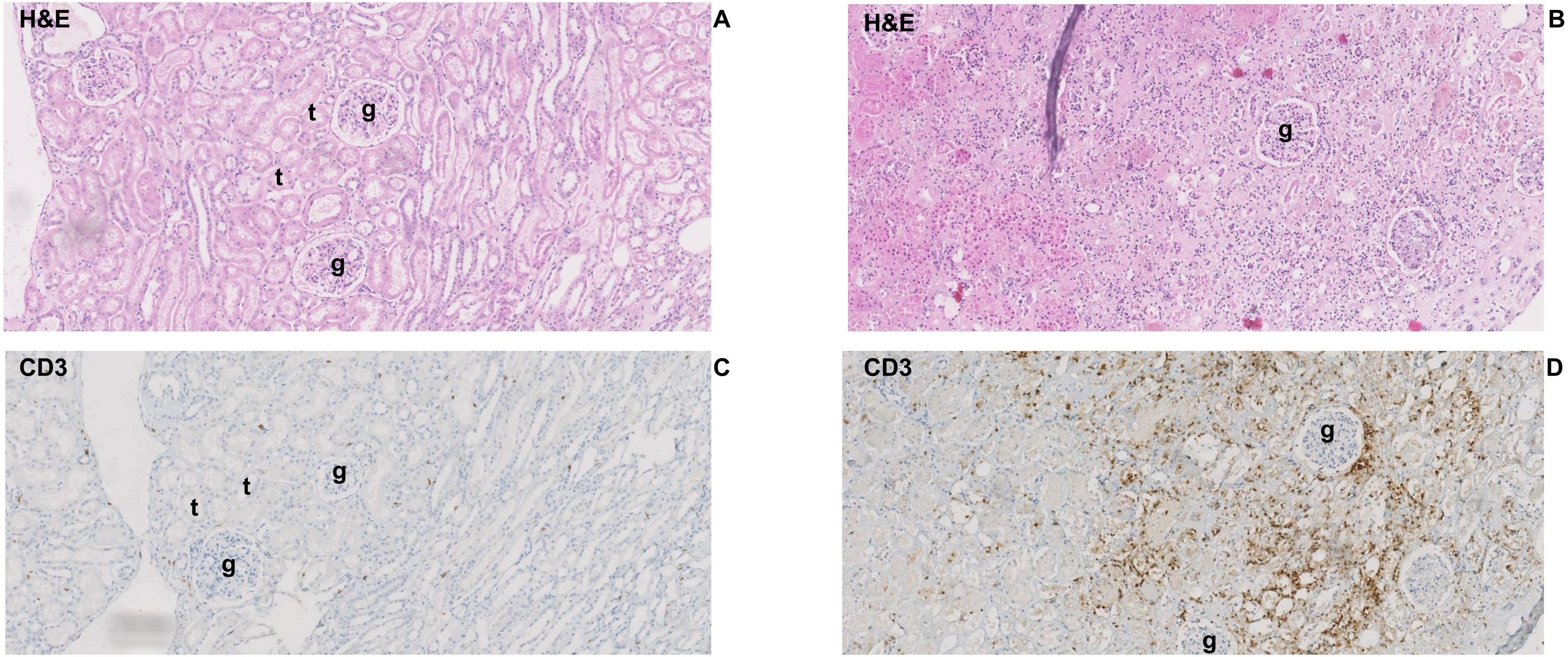

Supplementary Figure S1 Immunohistochemistry of kidney tissue

Healthy kidney tissue obtained via a nephrectomy was stained for hematoxylin and eosin (H&E; 10x magnification; A and B) and CD3 (10x magnification; C and D) before (A and C) and following four weeks of kidney tissue culture in presence of exogenous IL-2/IL-15 (B and D). A representative example is shown for in total 6 kidney tissues, 3 kidney tissues were obtained following a nephrectomy and 3 following a transplantectomy. Before culture, glomeruli (g) as well as tubuli (t) are observed and, the glomeruli, but not tubuli, stay intact following a four week explant culture. Interestingly, whereas hardly any T cells are present before culture (C, brown dots), numerous CD3+ T cells are present in the interstitium upon four weeks of culture in presence of exogenous IL-2/IL-15.
